# Human Dental Pulp Cells Responses to Apatite Precipitation from Dicalcium Silicates

**DOI:** 10.3390/ma8074491

**Published:** 2015-07-20

**Authors:** Wei-Yun Lai, Yi-Wen Chen, Chia-Tze Kao, Tuan-Ti Hsu, Tsui-Hsien Huang, Ming-You Shie

**Affiliations:** 1 School of Dentistry, Chung Shan Medical University, Taichung City 40447, Taiwan; E-Mails: joe1112joe@hotmail.com (W.-Y.L.); ctk@csmu.edu.tw (C.-T.K.); 2 Department of Stomatology, Chung Shan Medical University Hospital, Taichung City 40447, Taiwan; 3 3D Printing Medical Research Center, China Medical University Hospital, Taichung City 40447, Taiwan; E-Mail: evinchen@gmail.com; 4 Institute of Oral Science, Chung Shan Medical University, Taichung City 40447, Taiwan; E-Mail: nakocat86@yahoo.com.tw

**Keywords:** calcium silicate cement, apatite precipitated, human dental pulp cell, cell adhesion, fibronectin, collagen

## Abstract

Unraveling the mechanisms behind the processes of cell attachment and the enhanced proliferation that occurs as a response to the presence of calcium silicate-based materials needs to be better understood so as to expand the applications of silicate-based materials. Ions in the environment may influence apatite precipitation and affect silicate ion release from silicate-based materials. Thus, the involvement of apatite precipitate in the regulation of cell behavior of human dental pulp cells (hDPCs) is also investigated in the present study, along with an investigation of the specific role of cell morphology and osteocalcin protein expression cultured on calcium silicate (CS) with different Dulbecco’s modified Eagle’s medium (DMEM). The microstructure and component of CS cement immersion in DMEM and P-free DMEM are analyzed. In addition, when hDPCs are cultured on CS with two DMEMs, we evaluate fibronectin (FN) and collagen type I (COL) secretion during the cell attachment stage. The facilitation of cell adhesion on CS has been confirmed and observed both by scanning with an electron microscope and using immunofluorescence imaging. The results indicate that CS is completely covered by an apatite layer with tiny spherical shapes on the surface in the DMEM, but not in the P-free DMEM. Compared to the P-free DMEM, the lower Ca ion in the DMEM may be attributed to the formation of the apatite on the surfaces of specimens as a result of consumption of the Ca ion from the DMEM. Similarly, the lower Si ion in the CS-soaked DMEM is attributed to the shielding effect of the apatite layer. The P-free DMEM group releases more Si ion increased COL and FN secretion, which promotes cell attachment more effectively than DMEM. This study provides new and important clues regarding the major effects of Si-induced cell behavior as well as the precipitated apatite-inhibited hDPC behavior on these materials.

## 1. Introduction

Calcium silicate-based materials are used as a root-end dental filling material and are widely used in several endodontic clinical applications, such as in Mineral trioxide aggregate (MTA) [1], Biodentin [2], and Bioaggregate [3]. In dentistry, calcium silicate-based cements have been formulated into dentin restorative and replacement materials [4], but there is reason to believe their performance can be made more effective. For example, the handling properties can be improved and the setting time in clinical uses can be decreased [5]. Therefore, we have developed a fast setting calcium silicate (CS) cement that contains CaO, SiO_2_, and Al_2_O_3_, which have been demonstrated to reduce setting time in our lab [6]. We have demonstrated that CS cement not only exhibits good osteogenic effects [7,8], but also inhibits inflammation markers in primary human dental pulp cells (hDPCs) [9,10] and *in vivo* [11]. The Si ion released from silicate-based materials affects the behavior of different cell types by inhibiting osteoclastgenesis in macrophage [12], and the angiogenesis in hDPCs [13,14]. Moreover, Si concentration can affect various extracellular matrices (ECM) such as collagen I, fibronectin, and vitronectin adsorption on substrates and enhance the up-regulation of mitogen-activated protein kinase/extracellular signal-regulated protein kinase 1/2 (MAPK/ERK 1/2) via the calcium channel [15,16,17]. In a previous study, Zhou *et al.* proved the possible mechanism for the markedly stimulatory effect of CS-based materials extracts on the cementogenic differentiation of cells is related to the activation of the Wnt/b-catenin signaling pathway [18,19]. In addition, CS-based materials induced higher alkalinization of the environment and showed a higher bacteriostatic effect and growth inhibition towards *S. aureus* and *P. aeruginosa* than did calcium phosphate cement [20].

Silicate-based materials have gained increasing interest from researchers because of their high bioactivity [21,22]. As an example, calcium silicate cement has been shown to have excellent bone-like carbonated hydroxyapatite forming ability *in vitro* [23] and *in vivo* [11]. It is believed that the prerequisite for a bone substitute to bond to natural bone is the formation of a “bone-like” apatite layer, an indicator of bioactivity (the ability to form a chemical bond with living tissue). In a recent study, we developed a calcium silicate (CS) cement with high bioactivity which is able to precipitate an apatite layer on a substrate surface after soaking in a simulated body fluid (SBF) [21,24]. The bioactivity of these silicate-based materials indicates that the presence of PO_4_^3−^ ions in the composition is not an essential requirement for the development of an apatite layer, which consumes calcium and phosphate ions. This is because PO_4_^3−^ ions originate from the *in vitro* assay solutions [25]. 

Taking these factors together, we hypothesize that ions from the environment may influence apatite precipitation and affect Si ion release. Thus, the roles of apatite in the regulation of the cell behavior of hDPCs are also investigated in the present study, which focuses on an examination of the specific role of cell morphology and osteocalcin protein expression cultured on CS in DMEM with/without the P ion. 

## 2. Materials and Methods

### 2.1. Specimen Preparation

The CS cement used in this research was made according to our previously reported laboratory procedures [12]. First, appropriate amounts of CaO (65%, Sigma-Aldrich, St. Louis, MO, USA), SiO_2_ (25%, High Pure Chemicals, Saitama, Japan), and Al_2_O_3_ (5%, Sigma-Aldrich) powders were mixed. After sintering at 1400 °C for 2 h, the granules were ball milled in 99.5% EtOH using a centrifugal ball mill (Retsch S 100, Hann, Germany) for 6 h and then dried at 120 °C in an oven for 12 h. After mixing with H_2_O, the cements were molded in a Teflon mold (diameter: 6 mm, height: 3 mm). The cement quantities in our experiment were sufficient to fully cover each well of the 24-well plate (GeneDireX, Las Vegas, NV, USA) to a thickness of 2 mm for the cell experiments. All samples were stored in an incubator at 100% relative humidity at 37 °C for 1 day. 

### 2.2. Apatite Precipitate

To evaluate the *in vitro* bioactivity of the specimens, they were immersed in a 10 mL normal Dulbecco’s modified Eagle’s medium (DMEM) and phosphate-free DMEM (P-free DMEM) at 37 °C. Before soaking in DMEM, all the specimens were sterilized by immersion in 75% ethanol followed by exposure to an ultraviolet (UV) light for 1 h. After soaking for various time durations ranging from 3 to 24 h, the specimens were removed from the tube. The phase composition of the cements was then analyzed using X-ray diffractometry (XRD; Bruker D8 SSS, Karlsruhe, Germany) operated at 30 kV and 30 mA at a scanning speed of 1°/min. The morphology of the cement specimens was then examined under a scanning electron microscope (SEM; JSM-6700F, JEOL, Tokyo, Japan) operated in the lower secondary electron image (LEI) mode at 3 kV acceleration voltage.

### 2.3. Ion Concentration

The Ca, Si, and P ion concentrations released from the CS on the different DMEMs were determined using an inductively coupled plasma-atomic emission spectrometer (ICP-AES; Perkin-Elmer OPT 1MA 3000DV, Shelton, CT, USA) after the samples had been immersed for specific periods of time. Three samples were then measured for each data point, allowing the results to be obtained in triplicate from three separate samples for each test.

### 2.4. HDPCs Isolation and Culture

The hDPCs were freshly derived from caries-free, intact premolars that had been extracted for orthodontic treatment purposes, as described previously [10]. The patient gave informed consent, and approval from the Ethics Committee of the Chung Shan Medicine University Hospital was obtained (CSMUH No. CS14117). The tooth was then split sagittally with a chisel. The pulp tissue was immersed in phosphate-buffered saline (PBS; Caisson, North Logan, UT, USA) solution and digested in 0.1% collagenase type I (Sigma-Aldrich) for 30 min. After being transferred to a new culturing dish, the cells’ suspension was cultured in Dulbecco’s modified Eagle medium (DMEM; Caisson), containing 20% fetal bovine serum (FBS; GeneDireX), 1% penicillin (10,000 U/mL)/streptomycin (10,000 mg/mL) (PS, Caisson) and was kept in a humidified atmosphere with 5% CO_2_ at 37 °C. The medium was changed every 3 days. The hDPCs were subcultured through successive passaging at a 1:3 ratio until they were used for experiments (passages 3–8).

### 2.5. COL and FN Secretion

Cells were cultured on CS in different DMEMs for 1, 3, and 6 h, and the cell culture media were then collected and stored at room temperature. Human COL and FN enzyme linked immunosorbent assay (ELISA) kits were obtained from Abcam (Abcam, Cambridge, MA, USA). Following the manufacturer’s instructions we used a 3 h assay, which has a high degree of sensitivity. The reaction was terminated by the addition of a stop solution and read at 450 nm using a multiwell spectrophotometer. 

### 2.6. Cell Adhesion and Proliferation Assay

The suspended cells were kept at a density of 1.5 × 10^4^/specimen that were directly seeded over each sample in different DMEMs, after which the cell cultures were incubated at 37 °C in a 5% CO_2_ atmosphere. After being cultured for various pre-determined lengths of time (3, 6, 12 h, and 1, 3, 7 days), cell adhesion ability was evaluated using the PrestoBlue^®^ assay (Invitrogen, Grand Island, NY, USA). To describe the process briefly, each specimen was filled at a 1:9 ratio of PrestoBlue^®^ in fresh DMEM and incubated at 37 °C for 30 min. The solution in each well was then transferred to a new 96-well plate and read using a multiwell spectrophotometer (Hitachi, Tokyo, Japan) at 570 nm with a reference wavelength of 600 nm. The results were obtained in triplicate from three separate experiments for each test. Cells cultured on tissue culture plates without cement were used as a control (Ctl).

### 2.7. Cell Morphology

After the cells had been seeded for 3 h, the samples were washed three times with cold PBS and fixed in 1.5% glutaraldehyde (Sigma) for 2 h. The specimens were then dehydrated using a graded ethanol series for 20 min at each concentration and dried with liquid CO_2_ using a critical point dryer device (LADD 28000; LADD, Williston, VT, USA). The dried specimens were mounted on stubs, coated with gold, and viewed using SEM. For immunofluorescent staining, cells were seeded on CS in different DMEMs for the same time period. Cells were fixed using 4% paraformaldehyde for 30 min and permeabilized using 0.1% Triton X-100 for 15 min, following which the specimens were blocked with 2% bovine serum albumin (BSA) for 1 h and then incubated with AlexaFluor-594-conjugated phalloidin (F-actin, red) for 1 h at room temperature. The nuclei were then stained with DAPI (4′,6-diamidino-2-phenylindole, dilactate) for 1 h at room temperature. Finally, the samples were washed with TBS-T three times, and the cells were photographed under indirect immunofluorescence using a Zeiss Axioskop 2 microscope (Carl Zeiss, Thornwood, NY, USA).

### 2.8. Immunofluorescence Staining

For the immunofluorescent staining, cells were seeded on CS in different DMEMs for 7 days. The cells were fixed using 4% paraformaldehyde for 30 min and permeabilized by using 0.1% Triton X-100 for 15 min, blocked with 2% BSA for 1 h, incubated with the osteocalcin (OC) primary antibody at 4 °C overnight, washed, and then incubated with AlexaFluor-488-conjugated secondary antibodies (Invitrogen) (Green) for 1 h at room temperature. To detect the nucleus and actin filaments (F-actin), the cells were stained with DAPI (4′,6-diamidino-2-phenylindole, dilactate) (Invitrogen) and phalloidin conjugated with AlexFluor 594 (Invitrogen) (Green color) for 1 h at room temperature. The samples were then washed with TBS-T three times following which the cells were photographed under indirect immunofluorescence using a Zeiss Axioskop 2 microscope (Zeiss, Oberkochen, Germany).

### 2.9. Osteocalcin Formation

The osteocalcin (OC) protein released from the pulp cells was cultured on different substrates for 3 and 7 days after cell seeding. An OC enzyme-linked immunosorbent assay kit (Invitrogen) was used to determine OC protein content following the manufacturer’s instructions. The OC protein concentration was measured by correlation using a standard curve. The analyzed blank disks were treated as controls. All experiments were done in triplicate.

### 2.10. Statistical Analysis

A one-way variance statistical analysis was used to evaluate the significance of the differences between the groups in each experiment. Scheffe’s multiple comparison test was used to determine the significance of the deviations in the data for each specimen. In all cases, the results were considered statistically significant with a *p* value < 0.05.

## 3. Results

### 3.1. Characterization of CS in Different DMEM

**Figure 1:**
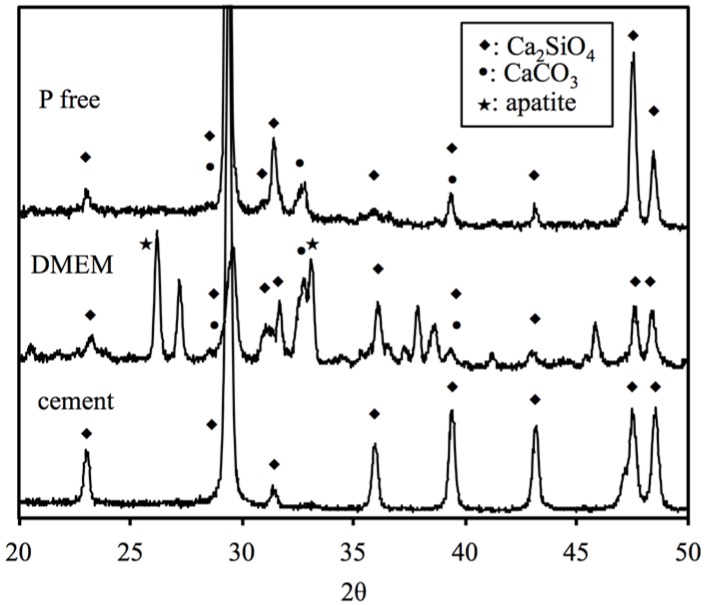
XRD patterns of CS before and after immersion in different DMEM for 24 h.

**Figure 2:**
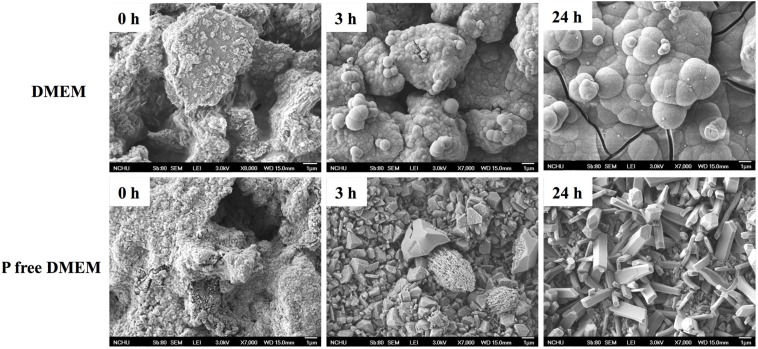
SEM micrographs of the CS surfaces before and after immersion in DMEM and P-free DMEM for different time points.

The XRD patterns of the CS before and after soaking in different DMEM for 1 day can be seen in Figure 1. The CS analysis reveals two key points: first, an obvious diffraction peak near 2θ = 29.4°, which corresponds to the calcium silicate hydrate (CSH) gel, and second, incompletely reacted inorganic component phases of the β-dicalcium silicate (β-Ca_2_SiO_4_) at 2θ between 32° and 34°. After soaking in DMEM, broad and diffuse peaks at 2θ = 25.9° and 31.8–32.9° clearly appear in the resulting XRD patterns, which may be ascribed to the characteristic peaks of apatite. In contrast, there is no apatite peak expression in the CS after immersion in P-free DMEM. Interestingly, the CaCO_3_ phases expressed with the use of P-free DMEM. The surface microstructure of the CS before and after immersion in different DMEM for 3 and 24 h, respectively are shown in Figure 2. The CS cement exhibits a dense and smooth surface containing particle entanglement and micro-pores before immersion. After immersion in DMEM for 3 and 24 h, the surface of CS becomes completely covered by an apatite layer with tiny spherical shapes on the surface. However, it is clear that no sphere precipitate has formed on the CS surfaces immersed in P-free DMEM.

### 3.2. Ion Concentration

**Figure 3:**
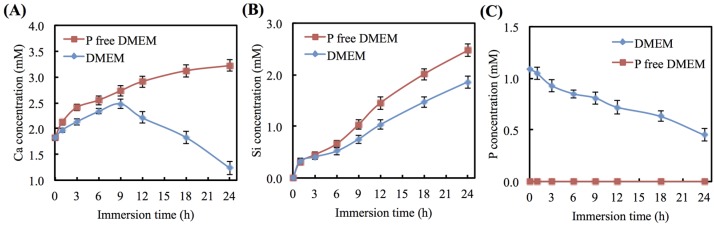
(**A**) Ca, (**B**) Si, and (**C**) P ion concentrations of CS after immersion in different DMEM for various time points.

The variations in the different DMEM Ca, Si, and P ion concentrations as measured at different times during the period after immersion are shown in Figure 3. The Ca ion concentration of the medium increased after being cultured for 9 h and then went to levels higher than the baseline Ca concentration of P-free DMEM (2.73 mM) (*p* < 0.05). A significant difference (*p* < 0.05) in the Ca ion concentration levels can be seen between P-free DMEM and DMEM after immersion for 9 h (*p* < 0.05). After 24 h, the Si ion concentration was approximately 2.5 and 1.9 mM at P-free DMEM and DMEM, respectively (Figure 3B). The P ion concentration of DMEM decreased after immersion, and the concentration ended up at approximately 0.45 mM (Figure 3C). There was no P ion detected in the P-free DMEM. 

### 3.3. COL and FN Secretion

**Figure 4:**
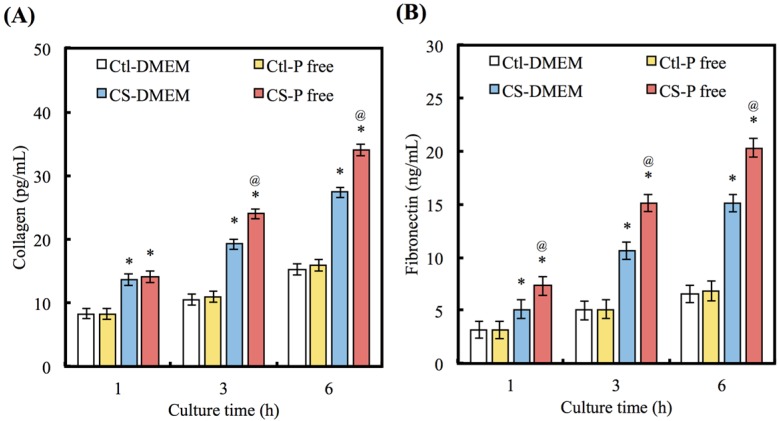
(**A**) COL and (**B**) FN were secret from hDPCs after seeding on CS with different DMEM for 1, 3, and 6 h. “*” indicates a significant difference (*p* < 0.05) compared to same material with DMEM. “@” indicates a significant difference (*p* < 0.05) compared to Ctl in same medium.

Figure 4A,B show the amounts of COL and FN protein in the culture medium secreted from cells cultured on CS with different DMEMs. At 1 h, the COL secretion from cells cultured on the CS is 1.64 (DMEM) and 1.71 (P-free DMDM) times higher (*p* < 0.05) than on Ctl. There are no significant differences (*p* > 0.05) between cells cultured on Ctl with different DMEMs. After 3 h, the value of the COL secretion from cells on CS with the P-free DMEM is significantly (*p* < 0.05) higher than for the DMEM. The results of FN secretion are similar to COL. 

### 3.4. Cell Adhesion and Proliferation

**Figure 5:**
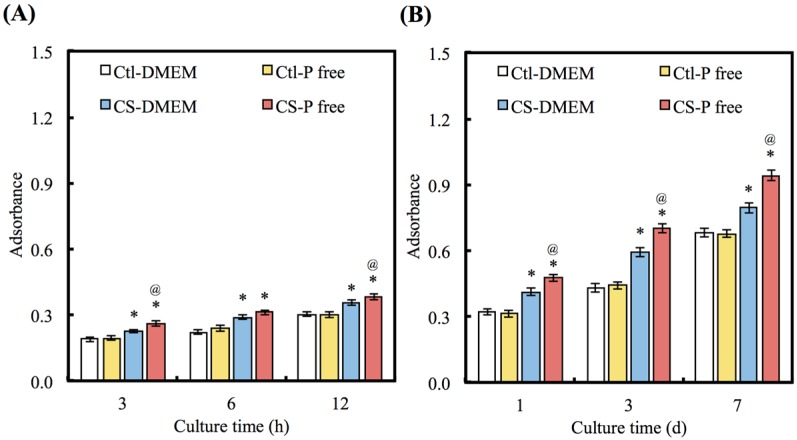
(**A**) The adhesion and (**B**) proliferation of hDPCs cultured with various DMEM for different time points. “*” indicates a significant difference (*p* < 0.05) compared to same material with DMEM. “@” indicates a significant difference (*p* < 0.05) compared to Ctl in same medium.

To consider the effects of apatite precipitate on cell adhesion and proliferation, the biological functions of hDPCs cultured on CS with different DMEMs were evaluated after different periods of time (Figure 5A,B). The Ctl group shows no significant differences in the ability of cell adhesion and proliferation (*p* > 0.05) between DMEM and P-free DMEM for all time points. Interestingly, there is a greater number of cells on the CS with the P-free DMEM than there is on the CS with the DMEM at all time-points (*p* < 0.05). The number of cells cultured on CS with the P-free DMEM shows an increase of 15%, 18%, and 20% in comparison with the DMEM on days 1, 3, and 7 of cell seeding, respectively.

### 3.5. Cell Morphology

**Figure 6:**
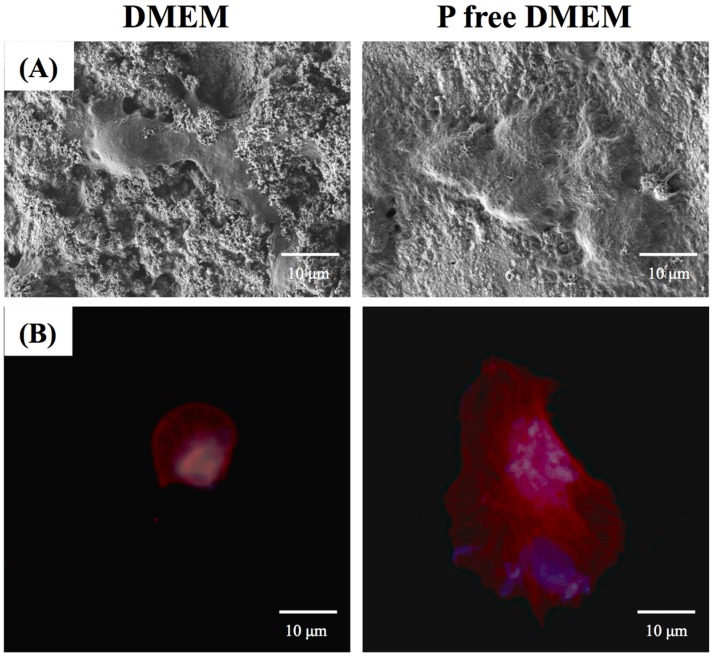
(**A**) SEM and (**B**) immunofluorescence images of hDPCs seeding on CS with two DMEM for 3 h.

The facilitation of cell adhesion on CS using different DMEMs was confirmed and observed using SEM (Figure 6A) and immunofluorescence images (Figure 6B). When the hDPCs were seeded onto CS substrates with DMEM for 3 h, the cells barely adhered or spread, whereas the cells cultured on CS with P-free DMEM exhibited normal adhesion. Fluorescence staining shows that at the same time point, hPDLs cultured on CS with P-free DMEM clearly displays the F-actin stress fiber morphologies of the cells (Figure 6B). In contrast, cells on the CS with DMEM exhibit a rounded morphology.

### 3.6. Osteocalcin Expression

**Figure 7:**
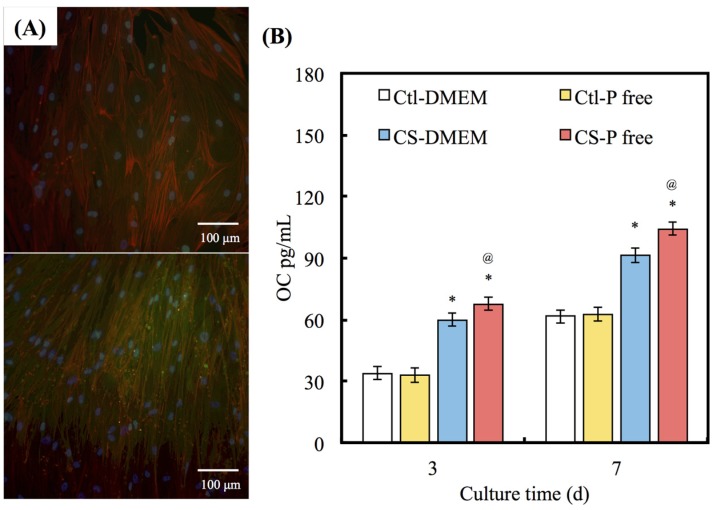
(**A**) Immunofluorescence images and (**B**) protein of OC expression of hDPCs cultured on CS with two DMEM for 7 days. (Nuclei: blue, F-actin: red and OC: green).

To further investigate the effects of apatite precipitate on hDPCs functions, OC was analyzed using immunofluorescence microscopy (Figure 7A). Visual examination shows that hDPCs cultured on the CS surfaces with P-free DMEM exhibit comparatively higher fluorescence intensity than those with DMEM after 7 days. Figure 7B shows the analysis of quantitative examination data and the OC activity of cells cultured on the different DMEMs for 3 and 7 days. The results show that OC levels increase in proportion with incubation time. A significant (13.1% and 15.2%) increase (*p* < 0.05) in the OC level was measured for CS in the P-free DMEM in comparison with the DMEM for 3 and 7 days, respectively. Interestingly, no significant differences (*p* > 0.05) were observable between the DMEM and the P-free DMEM in Ctl group for all time points.

## 4. Discussion

The products released from implant materials have been shown to affect cell behavior, including cell morphology, adhesion, proliferation, differentiation and mineralization [26,27]. The chemical composition of bone substitutes have been shown to affect cell behavior, including cell shape, attachment, proliferation, differentiation, and mineralized matrix synthesis [28,29]. CS materials have been proven to foster hDPCs adhesion, growth and differentiation and have been used as implant materials for hard tissue repair and regeneration. CS cement can be dissolved rapidly to release more Ca ions, thereby increasing the ionic activity of the apatite in the surrounding medium, which accelerates the nucleation rate of apatite precipitation [21]. Functional groups, such as Si–OH, on the surface of silicate-based materials have been shown to serve as nucleation centers for apatite precipitation. It has been documented that apatite is a potent regulator of cell behavior and has significant effects on the proliferation and differentiation of mesenchymal stem cells and osteoblasts [30]. In addition, several studies have conclusively shown that Si plays important roles in the early stages of bone formation and the calcification process [15,26]. Therefore, the efforts of the present research have been oriented toward unraveling those mechanisms using calcium silicate cements within different media cultured with hDPCs. Cell adhesion analysis reveals that the P-free DMEM is a more effective in regards to cell attachment and proliferation than DMEM, emphasizing the importance of the composition of the surrounding medium.

The bioactivity of the calcium-silicate based materials indicates that the presence of PO_3_^4−^ ions in the composition is not an essential requirement for the precipitation of the apatite layer on the material’s surface. This is noteworthy because it is known that PO_3_^4−^ consumes Ca and P ions because the PO_3_^4−^ ions originate from the immersion environment [21]. In addition, the Si–OH functional group on CS materials has been demonstrated to act as the nucleation center for apatite precipitation [11]. After soaking in normal DMEM, the formation of apatite spherulites was found on materials surfaces, which indicates high bioactivity of the current bone grafts. Thus, the CS-based cements will develop a stronger bond with natural bone tissue compared with other bone grafts. It is presumed that the reactions of partial dissolution and re-precipitation proposed for CS-based materials can exist when soaked in DMEM that represents PO_3_^4−^ source. After immersion, dissolution generally proceeds faster than apatite precipitation. When the precipitation process gets up to full speed, it eventually forms an apatite layer that effectively ‘‘seals’’ the surface underneath, after which the dissolution process stops. Compared to P-free DMEM, the lower Ca concentration in DMEM can be attributed to the formation of the apatite on the surfaces of cement specimens by consuming the Ca ion from DMEM. Similarly, the lower Si concentration in CS-soaked DMEM also seems to result from the shielding effect of the apatite layer.

Cell adhesion requires an appropriate proteinaceous substrate to which cell adhesion receptors, such as integrins, can adhere and form cell-anchoring points [31,32]. To elucidate the effects of apatite on osteogenic activity, the biological functions of pulp cells cultured on specimens were evaluated. The number of cells initially attached was different between the bone grafts with and without gelatin. Following initial adhesion, cells will secrete ECM components such as COL, FN, and vitronectin into the environment, which subsequently affect the cells’ behavior [10,33]. These proteins will adsorb on the material’s surface and supply a provisional matrix for cell adhesion. Cell adhesion requires the presence of an appropriate proteinaceous substrate to which cell adhesion receptors, such as integrins, can attach and form cell-anchoring points [10]. The dominating role of protein adsorption in the regulation of cell adhesion has been identified [15]. COL and FN are the main ECM molecules that are expressed and synthesized through all the stages of osteogenesis [34]. Differential ECM adsorption on the various material surfaces accounts for the observed variability in cell attachment [35]. Some studies have demonstrated the advantageous effects of Si on the stimulation of collagen secretion [26]. FN is the ECM molecule that is expressed and synthesized during the various stages of osteogenesis [36]. *In vivo*, the adsorption to the biomaterial surface of bioactive proteins from the serum and bodily fluids at the surgical site is known to influence cellular-material interactions [30]. Extracellular calcium ion is the major factor that affects cell adhesion and proliferation and has significant effects on the differentiation of cells, and the calcium ion has been known to involve in some proliferation signaling pathways. Silicate-based substrates, which release soluble silicate ions, have been shown to accelerate the formation of new bone tissue by promoting the gene and protein expression of osteogenic cells [16,37]. An appropriate Si concentration is effective in supporting the proliferation of hDPCs as well as actively stimulating their biological behavior through the production of osteogenic and angiogenic proteins [14,15]. The previous reports showed that bone incorporates various nutrients in the form of trace elements and Si had been found to play absolutely vital roles in the bone formation and regeneration and they are essential cofactors for enzymes that involve in the synthesis of the constituents of bone matrix [13,14]. However, the mechanism by which Si ion enhances cell behavior, including initial cell adhesion and proliferation, remains unclear. In other words, it is unclear which factors from Si contribute to its stimulation of osteogenesis and angiogenesis.

Cell-differentiation studies, like cell-adhesion and proliferation assay results, showed a significant impact of apatite, with an emphasis on the importance of material composition. OC protein was seen to increase up after 3 and 7 days on the materials, with protein increasing without apatite precipitation. It is generally accepted that an increase in OC in bone cells reflects a shift to a more differentiated state. OC is also associated with bone formation, and it is produced in high levels during the bone formation phase [38]. In the P-free DMEM group, the results suggest that CS releases a higher Si ion concentration that provides hDPCs with a more favorable microenvironment through enhanced adsorption of adhesion proteins; these proteins support cell adhesion and proliferation during the initial culture period. An increased level of cell adhesion would be expected to result in increased cell proliferation [39].

## 5. Conclusions

Unraveling the mechanisms behind the processes of cell attachment and enhanced proliferation which occur as a response to the presence of calcium silicate-based materials needs to be better understood so as to expand the applications of silica-based materials. Our results suggest that apatite precipitates on the CS surface at 3 h in DMEM, but not in P-free DMEM. In P-free DMEM, CS materials enhance cell adhesion and release greater quantities of Si ions than is the case with normal DMEM. The increased proliferation and osteogenic differentiation of hDPCs cultured on CS with P-free DMEM show that it is beneficial for cell growth. This study provides new and important clues regarding both the major effects of Si-induced cell behavior and the precipitated apatite inhibited hDPCs behavior of these materials.
